# TIMP-3 mRNA expression is regionally increased in moderately and poorly differentiated colorectal adenocarcinoma.

**DOI:** 10.1038/bjc.1997.285

**Published:** 1997

**Authors:** D. G. Powe, J. L. Brough, G. I. Carter, E. M. Bailey, W. G. Stetler-Stevenson, D. R. Turner, R. E. Hewitt

**Affiliations:** Department of Histopathology, Queen's Medical Centre, Nottingham, UK.

## Abstract

**Images:**


British Journal of Cancer (1997) 75(11), 1678–1683
© 1997 Cancer Research Campaign

### TIMP-3 mRNA expression is regionally increased in moderately and poorly differentiated colorectal adenocarcinoma

DG Powe[1], JL Brough[1], GI Carter[1], EM Bailey[1], WG Stetler-Stevenson[2], DR Turner[1] and RE Hewitt[1,2]

[1]Department of Histopathology, Queen's Medical Centre, Nottingham NG7 2UH, UK; [2]Laboratory of Pathology, National Cancer Institute, National Institutes of Health, Bethesda MD 20892-1500, USA

**Summary** In this study, we report on the distribution of tissue inhibitor of matrix metalloproteinase-3 (TIMP-3) mRNA expression in human normal colorectal mucosa, adenomas and adenocarcinomas. Northern blot analysis showed five TIMP-3 mRNA transcripts to be present in normal mucosal epithelium and in moderately and poorly differentiated carcinoma. Adenomas and well-differentiated carcinomas were not examined in this part of the investigation. In situ hybridization studies showed no detectable TIMP-3 mRNA in normal and adenomatous tissue. In contrast, TIMP-3 mRNA is localized to stromal fibroblast-like cells in colorectal carcinomas, with an increased incidence in moderately and poorly differentiated groups compared with well-differentiated carcinomas. Expression in both the moderately and the poorly differentiated tumour groups was strongest at the tumour invasive edge; none of the poorly differentiated carcinomas showed mRNA expression in regions ahead of the invasive edge, compared with 3 of 12 of the moderate group. To our knowledge, this is the first detailed report on the regional localization of TIMP-3 mRNA in colorectal tumours. We suggest that the lack of TIMP-3 mRNA expression in host stromal tissues ahead of poorly differentiated carcinomas may contribute to their increased invasiveness.

**Keywords**: colorectum; tissue inhibitor of metalloproteinases-3; in situ hybridization; Northern blotting analysis

Tumour invasion is a multistep process involving disruption of the epithelial basement membrane (EBM) and remodelling of the connective tissue matrix (Liotta and Stetler-Stevenson, 1991). A growing body of evidence supports the involvement of matrix metalloproteinases (MMPs) and their tissue inhibitors (TIMPs) in tumour invasion (Stetler-Stevenson, 1990; Hewitt et al, 1991; Poulsom et al, 1992). Three human TIMP forms have been described; TIMP-1 is a 28.5-kDa glycoprotein that inhibits interstitial collagenase (Docherty et al, 1985). TIMP-2 shares approximately 40% homology with TIMP-1 and inhibits type IV collagenase (Stetler-Stevenson et al, 1989). More recently, a third member, TIMP-3, has been described (Pavloff et al, 1992), having a predicted $M_r$ of 21.6 kDa (Uria et al, 1995) and is localized to chromosome 22 (Apte et al, 1994). TIMP-3 notably shares a high nucleotide homology (82.4%) with chicken inhibitor of metalloproteinase-3 (ChIMP3) (Pavloff et al, 1992) and shares a 25% amino acid homology with TIMP-1 and TIMP-2 (Apte et al, 1995). Inhibitory enzyme activities on the substrates human gelatinases A and B, collagenase-1 and stromelysin-1 are remarkably similar for TIMP-1 and TIMP-3 (Apte et al, 1995). Northern blot analysis has localized TIMP-3 mRNA in human breast tumours, placenta, uterus, heart, kidney, lung, pancreas, skeletal muscle and brain, but conflicting reports exist for its occurrence in liver (Apte et al, 1994; Uria et al, 1995). Apte et al (1994) propose that TIMP-3 has a regulatory role in the invasion of the uterus by the placental trophoblasts.

*Received 20 May 1996*
*Revised 27 November 1996*
Accepted 4 December 1996

*Correspondence to:* DG Powe

Previously, we demonstrated an association of TIMP-1 with the progression of colorectal tumours from adenomas to invasive adenocarcinoma (Hewitt et al, 1991); normal mucosa showed weak immunostaining while the adenomas showed an absence of TIMP-1 staining compared with the carcinomas. Davies et al (1993*a*) found TIMP-1 expression to be correlated to the more aggressive tumour phenotype in bladder cancers, while Zeng et al (1995*a*) identified increased TIMP-1 expression in colorectal carcinomas with metastases.

For the present study, we used in situ hybridization (ISH) and Northern blot analysis to investigate the cellular origin of TIMP-3 mRNA expression in normal mucosa, benign adenomas and adenocarcinoma of the colorectum. We also examined the relationship between the level of TIMP-3 expression and tumour grade.

### MATERIALS AND METHODS

### Tissue samples

Fresh, surgically resected bowel specimens received in the Histopathology Department, Queen's Medical Centre, were dissected with minimal delay to provide samples of normal mucosa, adenoma and adenocarcinoma. The adenocarcinoma were taken to include viable, non-necrotic tissue containing an invasive edge with adjacent normal mucosa. Tissue samples were divided into two portions: one piece was fixed in buffered 4% paraformaldehyde for 24 h before paraffin wax processing. The other sample was snap frozen in isopentane precooled in liquid nitrogen and stored at –70°C until required. Normal mucosa was dissected from uninvolved resection margins and treated similarly to the tumour samples. RNAase-free reagents and conditions were maintained.

Figure 1 This schematic diagram shows how the tumour sections were divided into regions for recording the TIMP-3 ISH results. The invasive edge (I) extended from the limit of visible tumour invasion to the edge of the tumour centre (TC), measuring two ×25 objective lens fields away. The adjacent host stroma (HA) region had the invasive edge as one border and extended two ×25 objective lens fields away to the border of the distant host stroma (HD)

Figure 2 Five transcripts were detected for TIMP-3 mRNA in colorectal normal mucosa (n) and adenocarcinoma (t); the strongest expression was seen in the 5.0-kb transcript. a = 5.0 kb, b = 2.1 kb, c = 1.6 kb, d = 1.4 kb, e = 1.0 kb

### Northern blotting

Total RNA was isolated from human colorectal tissue samples using the Trizol method (Life Technologies, Paisley, UK). Purity and yield of RNA were determined spectrophotometrically. For Northern blotting studies, 7 μg of each RNA sample was electrophoresed through 1% agarose–formaldehyde gel and blotted onto Hybond-N membrane (Amersham, Bucks, UK). Northern blot hybridization and autoradiography were as previously described with final stringency washes in 0.1 × SSPE/0.1% sodium dodecyl sulphate (SDS) (Hewitt et al, 1993).

### Probes

The human TIMP-3 plasmid was a gift from Dr J Uria and consisted of a 1.1-kb *Bam*HI/*Xho*I cDNA from a previously described plasmid (Uria et al, 1995) ligated into pBluescript SKII (Stratagene). Antisense cRNA probe was prepared by transcribing *Bam*HI linearized plasmid with T7 polymerase (Promega, Southampton, UK); sense cRNA probe was prepared by transcribing *Xho*I linearized plasmid with T3 polymerase. Probes were labelled with [$^{32}$P]CTP (Amersham, Bucks, UK) for Northern hybridization and [$^{35}$S]CTP (Du Pont, Herts, UK) for ISH. Probes used for ISH were hydrolysed to approximately 0.2 kb.

### TIMP-3 in situ hybridization

Paraffin wax-embedded sections (5 μm thick) were dewaxed in xylene and rehydrated to RNAase-free water. Using RNAase-free conditions throughout, the sections were treated according to a method based on that of Senior et al (1988). Sections were hybridized with a RNA probe at an activity equivalent to $1 \times 10^6$ c.p.m. per section. Subsequently, the sections were given stringency washes that included three 20-min washes in 60% formamide/4 × standard saline citrate (SSC) at 50°C, a wash in 2×SSC at 50°C and incubation in 100 μg $ml^{-1}$ RNAase A (Sigma, Dorset, UK) for 30 min at 37°C. Sections were washed in 0.1×SSC for 20 min at 37°C and then dehydrated in ethanol. Probe was detected using LM-1 autoradiographic emulsion (Amersham) with exposure for up to 24 days on replicate sections; sections were counterstained with haematoxylin and eosin.

Following in-situ hybridization, sections were subjectively assessed using bright- and dark-field illumination under low- and high-power magnifications. Each whole section was scanned for hybridization signal using low-power magnification (×10 objective) with dark-field illumination; areas showing increased signal were further investigated using high magnification (×40 objective) with bright-field illumination. Typically, 950 and 1250 cells were viewed per high-power field for stroma or stroma/neoplastic epithelium respectively. Cells showing more than 25 silver grains compared with the sense (negative control) were considered to show specific signal; replicate sections treated with the antisense and sense (negative control) probes were compared. The carcinoma sections were divided into the following regions and the presence of hybridization signal recorded: tumour centre (TC), tumour periphery (TP), neighbouring adjacent host (HA) stroma and distant host (HD) stroma (Figure 1).

### RESULTS

### Northern blot analysis

Twelve cases of paired tumour and uninvolved mucosa were analysed comprising nine moderately and three poorly differentiated adenocarcinomas. Following hybridization and exposure, the autoradiographs showed five bands on development (Figure 2); comparison with RNA standards estimated them to be 5.0 kb, 2.1 kb, 1.6 kb, 1.4 kb and 1.0 kb in size.

The optical densities (ODs) of the autoradiograph bands were determined using a Seescan (Cambridge, UK) image analyser and the tumour–normal ratios calculated (data not shown).

The 5.0-kb transcript showed a higher level of expression than the four smaller transcripts in 7 of 12 paired samples. TIMP-3 expression was increased in five of nine moderately differentiated tumours compared with the corresponding normal mucosa, with up to a threefold increase (range 1.07–3.2). In contrast, the remaining four moderately differentiated tumours showed

© Cancer Research Campaign 1997

**Table 1** In situ hybridization results for TIMP-3 mRNA in well (WD)-, moderately (MD) and poorly (PD) differentiated colorectal adenocarcinomas.

| Case number | Tumour grade | Cell type | Signal localization | | | |
|---|---|---|---|---|---|---|
| | | | TC | I | H Adj | H Dist |
| 1 | WD | NA | – | – | – | – |
| 2 | WD | NA | – | – | – | – |
| 3 | WD | NA | – | – | – | – |
| 4 | WD | F | – | Yes | – | – |
| 5 | WD | NA | – | – | – | – |
| 6 | MD | NA | – | – | – | – |
| 7 | MD | NA | – | – | – | – |
| 8 | MD | F | Yes | Yes | Yes | Yes |
| 9 | MD | NA | – | – | – | – |
| 10 | MD | F | – | Yes | – | – |
| 11 | MD | F | Yes | Yes | Yes | – |
| 12 | MD | NA | – | – | – | – |
| 13 | MD | F | – | Yes | – | – |
| 14 | MD | F | Yes | Yes | – | Yes |
| 15 | MD | F | – | Yes | – | – |
| 16 | MD | F | – | Yes | – | – |
| 17 | MD | NA | – | – | – | – |
| 18 | PD | F | Yes | Yes | – | – |
| 19 | PD | F | Yes | Yes | – | – |
| 20 | PD | F | – | Yes | – | – |
| 21 | PD | F | Yes | Yes | – | – |
| 22 | PD | F | – | Yes | – | – |
| 23 | PD | F | – | Yes | – | – |
| 24 | PD | NA | – | – | – | – |

F, fibroblast-like cell shows signal; NA, not applicable; TC, tumour centre; I, invasive edge; H Adj, host adjacent stroma; H Dist, host distant stroma; –, no signal detected.

increased expression in the matched normal samples (range 1.02–1.77). Slight increases in TIMP-3 expression occurred in two of three poorly differentiated tumours (range 1.15–1.19).

The four smaller transcripts of TIMP-3 (2.1 kb, 1.6 kb, 1.4 kb and 1.0 kb) showed increased expression in the normal mucosa samples (range 1.02–3.35) in all but two cases; one of the moderately differentiated tumours showed increased expression in all five transcripts (range 2.65–3.5), while a second tumour of the same grade showed increased expression in the 1.0-kb and 1.4-kb transcripts.

### In situ hybridization

A positive cellular ISH signal was determined by increased silver grain frequency per cell and by comparison of the antisense and sense (negative control) probed sections.

All five samples of uninvolved normal mucosa obtained from the surgical resection margins were negative for TIMP-3 mRNA. Additionally, many of the adenocarcinoma samples contained normal mucosa adjacent to the neoplastic epithelium and none showed specific signal. Also, none of the six adenoma samples investigated showed specific hybridization signal with the TIMP-3 antisense probe.

An increased number of silver grains were seen localized to stromal cells in only one of five well-differentiated bowel samples probed with the TIMP-3 antisense probe (Table 1). Many of the cells showing signal were spindle shaped or had large open nuclei and were most probably fibroblasts, though the involvement of macrophages or endothelial cells can not be entirely discounted. Messenger RNA expression was localized focally at the tumour invasive edge.

In marked contrast, 7 of 12 cases of the moderately differentiated tumours showed TIMP-3 expression (Table 1) localized to stromal cells (Figure 3A, B). All of the positive cases showed localization at the invasive edge, with the distribution in the adjacent host stroma being the second most frequent site; signal in distant host stroma was seen in some cases. The poorly differentiated adenocarcinomas showed TIMP-3 mRNA expression in fibroblast-like cells at the invasive edge of six out of seven cases examined (Table 1; Figure 3C); three of these cases showed signal in stromal cells in the tumour centre. None of the cases analysed showed signal in stromal cells ahead of the invasive edge and, generally, the signal was weaker than that seen for the moderately differentiated group of carcinomas.

Replicate sections probed with the labelled-sense probe (negative control) showed background levels of signal only, with grain distributed over all cell types (Figure 3D).

### DISCUSSION

MMPs and TIMPs are involved in normal developmental and pathological processes (Nomura et al, 1989; Firestein and Paine, 1992). The study of the distribution and biological mechanism of action of MMPs and TIMPs is proving to be of therapeutic importance (Davies et al, 1993*b*). In the current study, we present a detailed description of the regional localization for TIMP-3 mRNA in normal mucosa, adenomas and in well-, moderately and poorly differentiated adenocarcinomas of the bowel.

We identified five transcripts for TIMP-3 (5.0 kb, 2.1 kb, 1.6 kb, 1.4 kb and 1.0 kb) using Northern analysis, which is in agreement with the number identified by Uria et al (1995) in a study of breast

© Cancer Research Campaign 1997

**Figure 3** (**A**) A moderately differentiated colonic adenocarcinoma probed for TIMP-3 mRNA. Hybridization signal (arrowed) is distributed in the stroma surrounding the neoplastic glands (N) at the tumour invasive edge. **B** (Detail of **A**) and **C** (poorly differentiated adenocarcinoma) show that TIMP-3 mRNA is localized to round and spindle-shaped stromal cells (arrowed). (**D**) A replicate section of **B** treated with the TIMP-3 sense (negative control) probe showing background signal only. Counterstain: haematoxylin and eosin. Bar = 25 µm tumour, placenta and uterus. However, in the latter study, the transcripts were estimated to be 5.0 kb, 2.7 kb, 2.4 kb, 1.6 kb and 1.1 kb. Apte et al (1994) identified only three TIMP-3 transcripts (5.5 kb, 2.8 kb and 2.4 kb) in a comprehensive range of tissues, including kidney, lung and pancreas, but the 2.8-kb transcript was absent from brain.

In normal colonic mucosa, we identified predominantly elevated TIMP-3 mRNA expression in four of the five transcripts (2.1 kb, 1.6 kb, 1.4 kb and 1.0 kb). In contrast, 7 of 12 tumour cases showed increased expression of the largest (5.0 kb) transcript and, generally, this transcript gave a stronger band on autoradiography than the smaller ones, suggesting a higher abundance. Interestingly, in breast tumours, uterus and placenta, the 2.4-kb transcript appears to be overexpressed (Uria et al, 1995). Despite using the same probe as Uria et al (1995), the differences in transcript size and relative level of expression reported here may prove to be important. Uria et al (1995) suggest a number of explanations, including alternative polyadenylation sites, variable extension of the 5′ flanking region or the existence of additional genes possessing similar sequences to those of TIMP-3. Byrne et al (1995) proposed

© Cancer Research Campaign 1997

that cell and tissue factors are at least responsible for controlling the selection of the polyadenylation sites. Our findings add further to the evidence that tissue-specific factors influence the pattern of expression of the different TIMP-3 transcripts.

Northern analysis showed TIMP-3 mRNA expression in normal mucosa. However, using ISH, specific TIMP-3 mRNA expression was not detected in either normal colorectal mucosa or adenomas. An absence of discernible signal in the ISH-probed normal mucosa may be due to differences in signal to noise ratios between the two techniques. These findings are consistent with those of Zeng et al (1995*a*) who found that normal colonic mucosa did not express TIMP-1 mRNA by ISH. Our findings suggest a diffuse distribution of low level TIMP-3 expression in normal mucosa, which is detectable by Northern blotting but below the sensitivity of the ISH technique used. In situations of low mRNA abundance, ISH signal differences between antisense- and sense-probed sections may be difficult to interpret because of the annealing of vector sequences of sense RNA probes, generated using the pBluescript vector, annealing to human 28s rRNA (Witkiewicz et al, 1993).

We demonstrated stromal TIMP-3 mRNA expression in round and spindle-shaped cells with a fibroblast-like appearance, but the possibility that these cells were macrophages or endothelial cells cannot be excluded. TIMPs have been shown to be expressed in endothelial cells during angiogenesis (Cornelius et al, 1995). TIMP-3 expression was seen in moderately and poorly differentiated colorectal carcinomas with localization at the invasive edge being a feature of both groups. However, it is interesting and perhaps significant to note the localizational differences between the two tumour grades. Whereas strong expression is seen throughout the tumour and in adjacent and distant host stroma of moderately differentiated cancers, poorly differentiated carcinomas failed to show TIMP-3 localization in the stroma ahead of the invasive edge. Overall, a weaker ISH signal was seen with the poorly differentiated carcinomas, suggesting reduced levels of expression. Our findings corroborate those of Byrne et al (1995) who identified that the strongest TIMP-3 ISH signal in breast carcinomas occurred in fibroblasts closest to the neoplastic cells. Tumour-secreted factors acting over a short range may account for the occurrence of activated fibroblasts in this region. Previous immunochemical studies have localized a MMP-inducing factor to the peripheral tumour cells of transitional bladder cancers (Muraoka et al, 1993); TIMP-1 has also been associated with MMP inducement (Clark et al, 1994).

In vivo, MMPs and TIMPs may be independently expressed, such is the case with TIMP-1 and MMP-9 (Zeng and Guillem, 1995). The TIMP family of molecules have a highly conserved N-terminal structure with TIMP-3 and TIMP-1 sharing a 72% amino acid homology. Similarities in the N-terminal region probably account for the similar pattern of inhibition seen (Apte et al, 1995). Currently, the precise MMP substrates for TIMP-3 are unknown but if TIMP-3 shares similar inhibiting properties to TIMP-2, it may be involved in regulating EBM breakdown. Previous studies have demonstrated the localized distribution and temporal occurrence of MMPs and TIMPs. Matrilysin (MMP-7) has been implicated in basement membrane degradation and occurs in increased expression in the highly invasive colonic carcinomas (Newell et al, 1994). MMP-2 (synthetic gelatinase A) mRNA expression has been localized in fibroblasts at the invasive edge of colorectal cancer using ISH (Poulsom et al, 1992; Pyke et al, 1993), and active MMP-2 enzyme has been zymographically demonstrated to be correlated to increasing tumour grade in bladder tumours (Davies et al, 1993*a*).

Our findings support the proposal of tumour invasion being a result of an MMP–TIMP imbalance (Stetler-Stevenson, 1990). The importance of the regional localization of TIMP-3 mRNA expression, using the in situ hybridization technique, is seen in considering the moderately and poorly differentiated tumour groups. An absence of TIMP-3 ISH signal in the stroma ahead of the invasive edge was seen in the more aggressive poorly differentiated carcinomas. In these regions, unopposed MMP activity may account for the increased frequency of metastasis seen in this group of tumours. The MMP-inhibitory function of TIMP-1 was demonstrated in an in vitro study by Khokha et al (1989) in which TIMP antisense RNA-treated Swiss 3T3 fibroblasts had reduced TIMP-1 levels, resulting in increased tumour growth and metastasis. The increased TIMP-3 expression seen at the invasive edge of moderately and poorly differentiated carcinomas in the current study is probably explained by the net balance of MMP–TIMP, with MMP expression increased relative to that of the inhibitor. The decline in the TIMP-3 mRNA expression (for moderately and poorly differentiated tumours) with increasing distance from the tumour mass is consistent with our previous immunochemical study in which TIMP-1 staining was seen to be reduced with increasing distance from the tumour invasive edge (Hewitt et al, 1991).

Lack of detectable mRNA expression in the well-differentiated tumour group may be due to reduced TIMP-3 (and its MMP substrate) involvement in the early events of invasion, or differential expression of TIMP-3 may occur as the tumour loses its differentiation. Northern analysis was not performed on well-differentiated tumours in this study.

The localization of TIMP-3-expressing stromal cells is broadly similar to that for TIMP-1 in moderately differentiated carcinomas (Powe et al, in preparation), but the TIMP-3 mRNA expression was decreased compared with TIMP-1 in poorly differentiated cancers. Both inhibitors have been shown to have similar activities on the same substrates, including human gelatinase A and B, collagenase-1 and stromelysin-1 (Apte et al, 1995). In addition to their inhibition properties, TIMP-1 and TIMP-2 also have growth-stimulating properties (Docherty et al, 1985; Stetler-Stevenson et al, 1989) and TIMP-1 has been shown to increase fibroblast collagenase production (Clark et al, 1994). In the current study, maximal TIMP-3 expression occurred in the stroma associated with the tumour invasive edge of the moderately and poorly differentiated colorectal adenocarcinomas. Tumour growth-promoting properties of TIMPs may account for the increased invasiveness associated with this group of tumours and for the findings of Fong et al (1996). In non-small-cell lung carcinomas, Fong et al (1996) showed an association between high TIMP-1 expression and poor prognosis.

In summary, TIMP-3 expression is increased at the invasive edge of moderately and poorly differentiated adenocarcinomas compared with well-differentiated carcinomas, adenomas and normal mucosa. In addition, both tumour groups show markedly reduced expression in fibroblast-like cells ahead of the tumour invasive edge but important regional differences also exist between the two groups. Deficient TIMP-3 expression ahead of the invasive edge of poorly differentiated tumours may be associated with increased tumour invasion and metastasis due to unopposed MMP activity.

© Cancer Research Campaign 1997

### ACKNOWLEDGEMENTS

The authors thank Miss Anne Kane for her skilled photographic assistance. This project was funded by a grant from the Trent Regional Health Authority and Departmental funds.

### REFERENCES

Apte SS, Mattei MG and Olsen BR (1994) Cloning of the cDNA encoding human tissue inhibitor of metalloproteinase-3 (TIMP-3) and mapping of the TIMP-3 gene to chromosome 22. *Genomics* **19**: 86–90

Apte SS, Olsen BR and Murphy G (1995) The gene structure of tissue inhibitor of metalloproteinases (TIMP)-3 and its inhibitory activities define the distinct TIMP gene family. *J Biol Chem* **270**: 14313–14318

Byrne JA, Tomasetto C, Rouyer N, Bellocq JP, Rio MC and Basset P (1995) The tissue inhibitor of metalloproteinases-3 gene in breast carcinoma: identification of multiple polyadenylation sites and a stromal pattern of expression. *Mol Med* **4**: 418–427

Clark IM, Powell LK and Cawston TE (1994) Tissue inhibitor of metalloproteinases (TIMP-1) stimulates the secretion of collagenase from human skin fibroblasts. *Biochem Biophys Res Comm* **203**: 874–880

Cornelius LA, Nehring LC, Roby JD, Parks WC and Welgus HG (1995) Human dermal microvascular matrix metalloproteinases in response to angiogenic factors and migration. *J Invest Dermatol* **105**: 170–176

Davies B, Waxman J, Harpret W, Abel P, Williams G, Krausz T, Nezl D, Thomas D, Hanby A and Balkwill F (1993*a*) Levels of matrix metalloproteinases in bladder cancer correlate with tumour grade and invasion. *Cancer Res* **53**: 1–5

Davies B, Brown PD, East N, Crimmin MJ and Balkwill F (1993*b*) A synthetic matrix metalloproteinase inhibitor decreases tumour burden and prolongs survival of mice bearing human ovarian carcinoma xenografts. *Cancer Res* **53**: 2087–2091

Docherty AJP, Lyons A, Smith BJ, Wright EM, Stephens PE, Harris TJR, Murphy G and Reynolds JJ (1985) Sequence of human tissue inhibitor of metalloproteinases and its identity to erythroid-potentiating activity. *Nature* **318**: 66–69

Firestein GS and Paine MM (1992) Stromelysin and tissue inhibitor of metalloproteinases gene expression in rheumatoid arthritis synovium. *Am J Pathol* **140**: 1309–1314

Fong KW, Kida Y, Zimmerman PV and Smith PJ (1996) TIMP-1 and adverse prognosis in non-small cell lung cancer. *Clin Cancer Res* **2**: 1369–1372

Hewitt RE, Leach IH, Powe DG, Clark IM, Cawston TE and Turner DR (1991) Distribution of collagenase and tissue inhibitors of metalloproteinases (TIMP) in colorectal tumours. *Int J Cancer* **49**: 666–672

Hewitt RE, Powe DG, Carter GI and Turner DR (1993) Desmoplasia and its relevance to colorectal tumour invasion. *Int J Cancer* **53**: 62–69

Khokha R, Waterhouse P, Yagel S, Lala PK, Overall CM, Norton G and Denhardt DT (1989) Antisense RNA-induced reduction in murine TIMP levels confers oncogenicity on Swiss 3T3 cells. *Science* **243**: 947–950

Liotta LA and Stetler-Stevenson WG (1991) Tumour invasion and metastasis: an imbalance of positive and negative regulation. *Cancer Res* **51**: 5054s–5059

Muraoka K, Nabeshima K, Murayama T, Biswas C and Koono M (1993) Enhanced expression of a tumour-cell-derived collagenase stimulatory factor in urothelial carcinoma: its usefulness as a tumour marker for bladder cancers. *Int J Cancer* **55**: 19–26

Newell KJ, Witty JP, Rodgers WH and Matrisian LM (1994) Expression and localisation of matrix-degrading metalloproteinases during colorectal tumorigenesis. *Mol Carcinogen* **10**: 199–206

Nomura S, Hogan BLM, Wills A, Heath JK and Edwards DR (1989) Developmental expression of tissue inhibitor of metalloproteinase (TIMP) RNA. *Development* **105**: 575–583

Pavloff N, Staskus PW, Kishani NS and Hawkes SP (1992) A new inhibitor of metalloproteinases from chicken: ChIMP-3. A third member of the TIMP family. *J Biol Chem* **267**: 17321–17326

Poulsom R, Pignatelli M, Stetler-Stevenson WG, Liotta LA, Wright PA, Jeffrey RE, Longcoft JM, Rogers L and Stamp GWH (1992) Stromal expression of 72 kDa type IV collagenase (MMP-2) and TIMP-2 mRNAs in colorectal neoplasia. *Am J Pathol* **141**: 389–396

Pyke C, Ralfkiaer E, Tryggvason K and Dano K (1993) Messenger RNA for two type IV collagenases is located in stromal cells in human colon cancer. *Am J Pathol* **142**: 359–365

Senior PV, Critchley DR, Beck F, Walker R and Varley JM (1988) The localisation of laminin mRNA and protein in the postimplantation embryo and placenta of the mouse: an *in situ* hybridisation and immunochemical study. *Development* **104**: 431–446

Stetler-Stevenson WG (1990) Type IV collagenases in tumour invasion and metastasis. *Cancer Metastasis Rev* **9**: 289–303

Stetler-Stevenson WG, Krutsch HC and Liotta LA (1989) Tissue inhibitor of metalloproteinase (TIMP-2). *J Biol Chem* **264**: 17374–17378

Uria JA, Adolfo AF, Velasco G, Freije JM and Lopez-Otin (1995) Structure and expression in breast tumours of human TIMP-3, a new member of the metalloproteinase inhibitor family. *Cancer Res* **54**: 2091–2094

Witkiewicz H, Bolander ME and Edwards DR (1993) Improved design of riboprobes from pBluescript and related vectors for in situ hybridisation. *Biotechniques* **14**: 458–463

Zeng ZS, Cohen AM, Zhang ZF, Stetler-Stevenson WG and Guillem JG (1995*a*) Elevated tissue inhibitor of metalloproteinase-1 (TIMP-1) RNA in colorectal cancer stroma correlates with lymph node and distant metastasis. *Clin Cancer Res* **1**: 899–906

Zeng ZS and Guillem JG (1995*b*) Distinct patterns of matrix metalloproteinase 9 and tissue inhibitor of metalloproteinase 1 mRNA expression in human colorectal cancer and liver metastases. *Br J Cancer* **72**: 575–582

© Cancer Research Campaign 1997


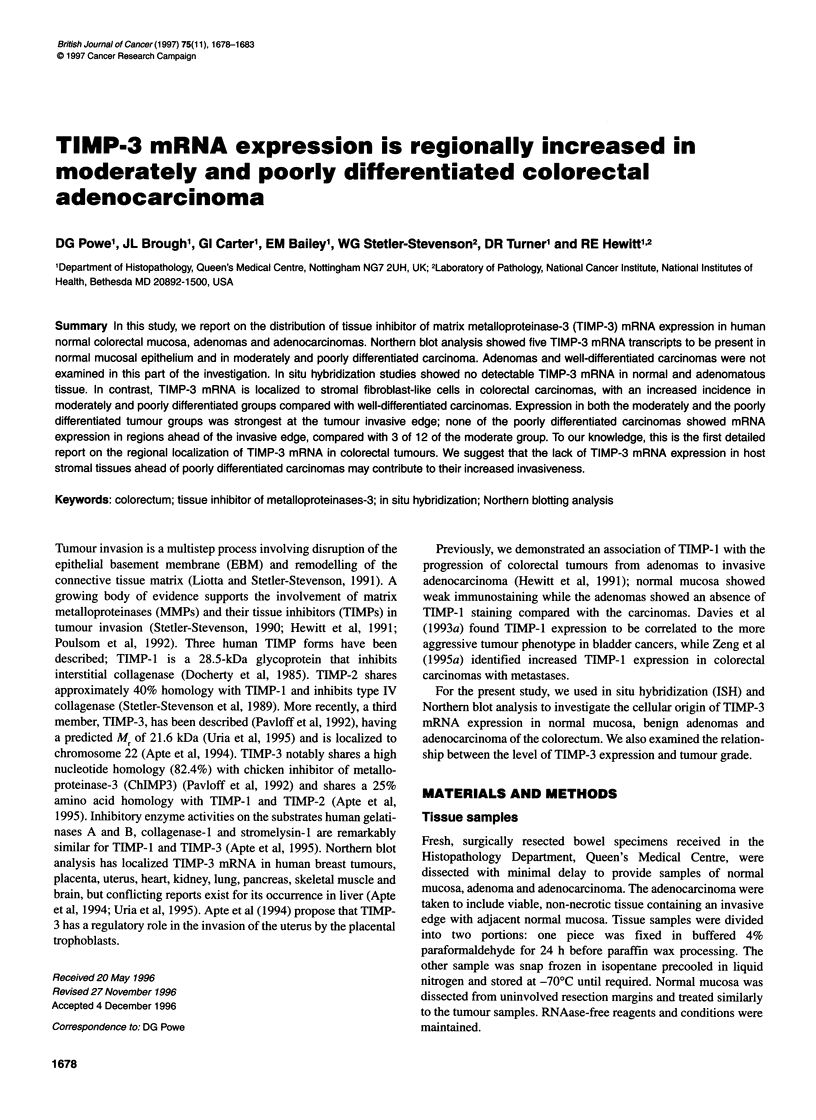

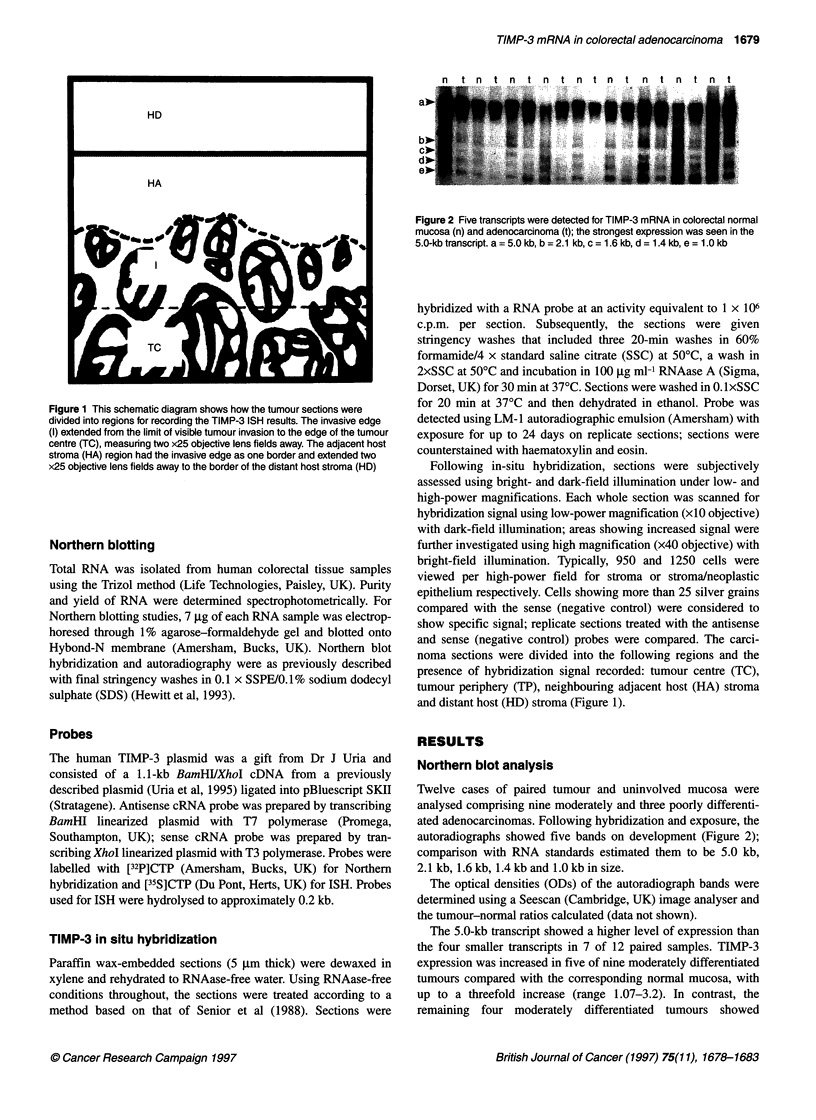

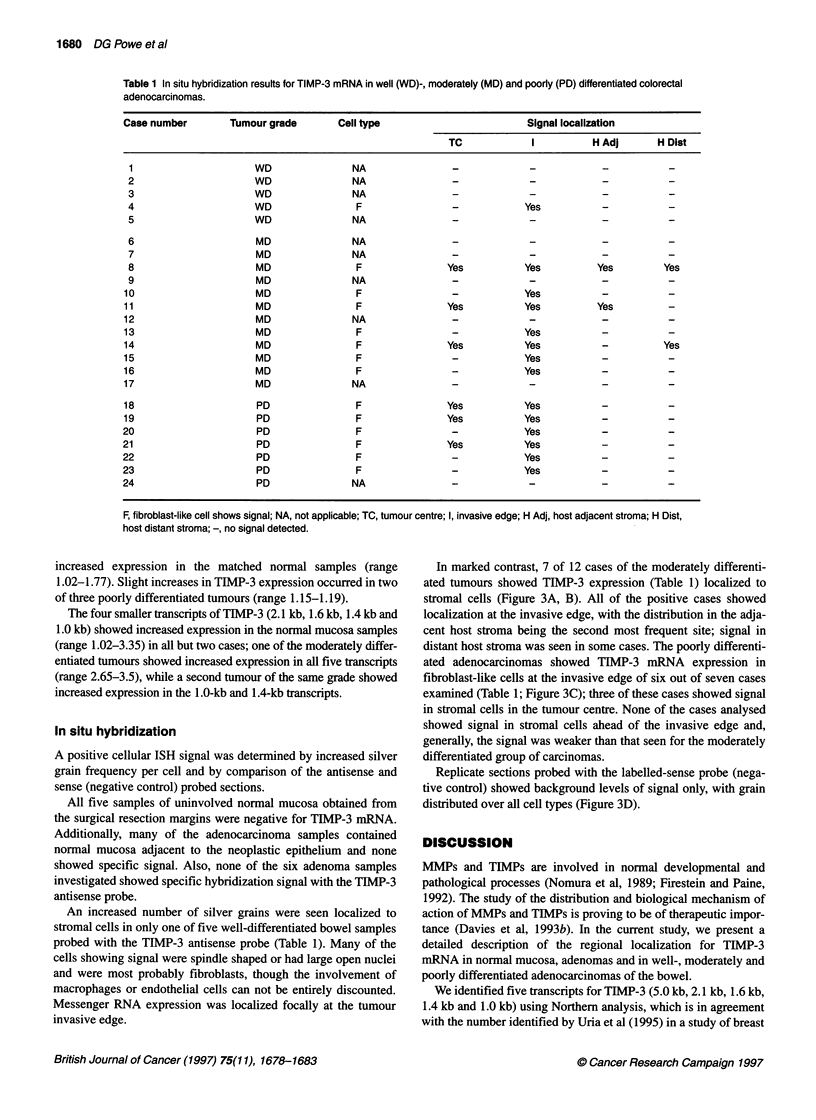

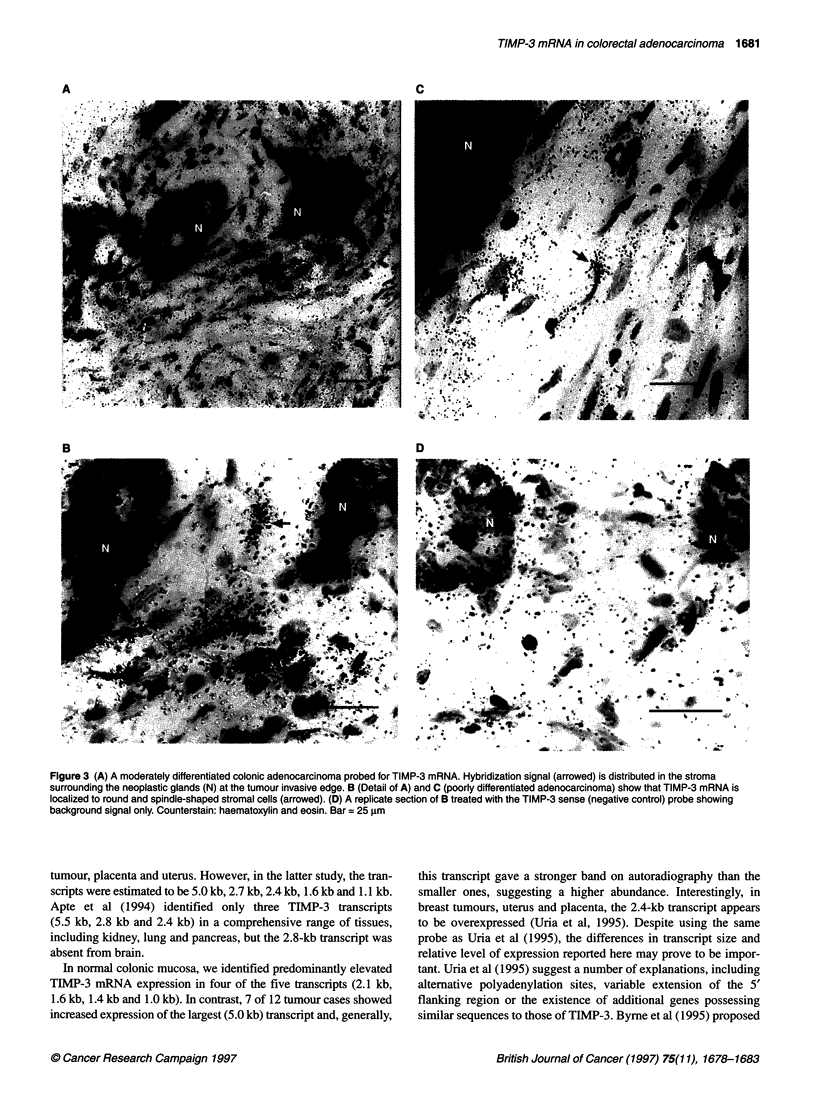

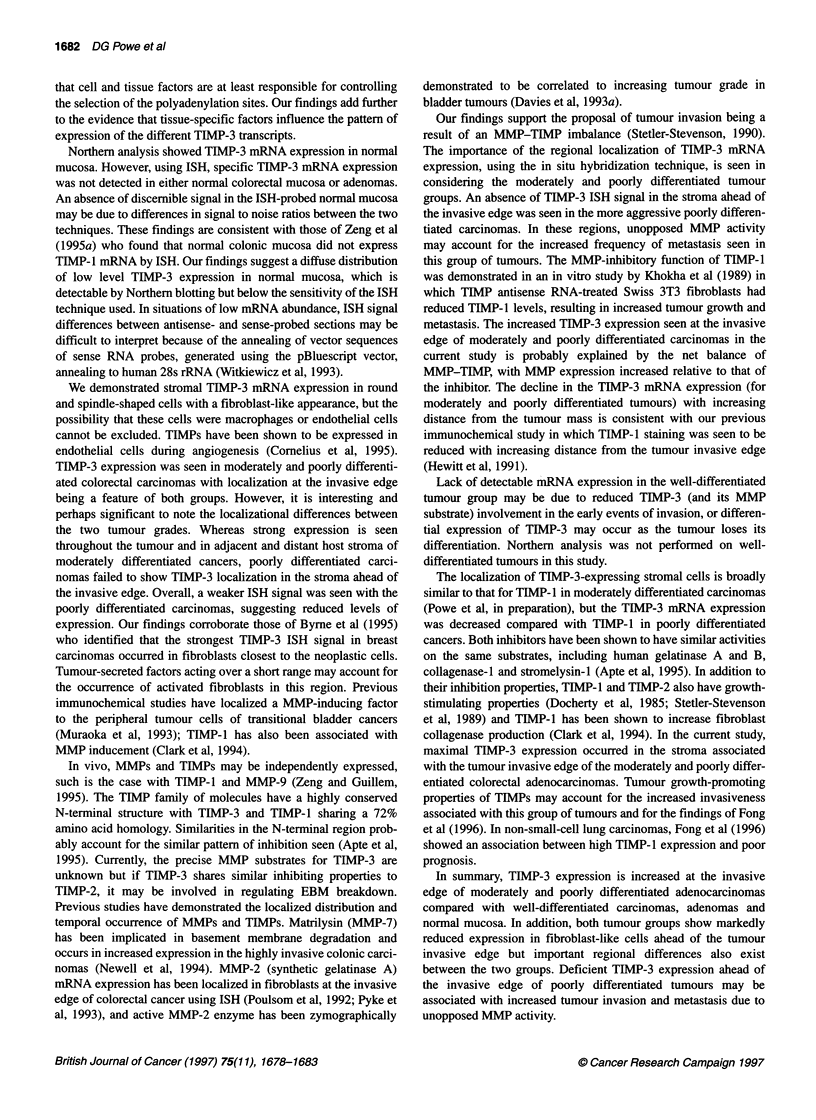

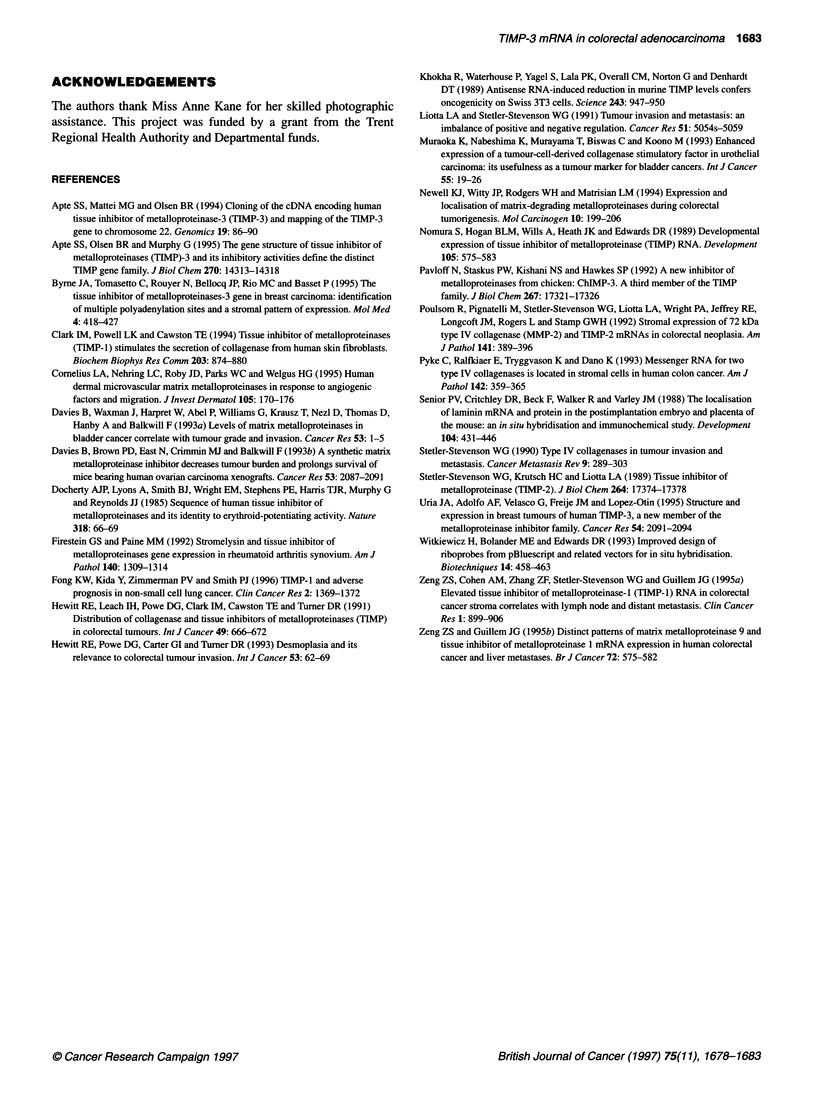


## References

[OCR_00512] Apte S. S., Mattei M. G., Olsen B. R. (1994). Cloning of the cDNA encoding human tissue inhibitor of metalloproteinases-3 (TIMP-3) and mapping of the TIMP3 gene to chromosome 22.. Genomics.

[OCR_00517] Apte S. S., Olsen B. R., Murphy G. (1995). The gene structure of tissue inhibitor of metalloproteinases (TIMP)-3 and its inhibitory activities define the distinct TIMP gene family.. J Biol Chem.

[OCR_00522] Byrne J. A., Tomasetto C., Rouyer N., Bellocq J. P., Rio M. C., Basset P. (1995). The tissue inhibitor of metalloproteinases-3 gene in breast carcinoma: identification of multiple polyadenylation sites and a stromal pattern of expression.. Mol Med.

[OCR_00528] Clark I. M., Powell L. K., Cawston T. E. (1994). Tissue inhibitor of metalloproteinases (TIMP-1) stimulates the secretion of collagenase from human skin fibroblasts.. Biochem Biophys Res Commun.

[OCR_00533] Cornelius L. A., Nehring L. C., Roby J. D., Parks W. C., Welgus H. G. (1995). Human dermal microvascular endothelial cells produce matrix metalloproteinases in response to angiogenic factors and migration.. J Invest Dermatol.

[OCR_00544] Davies B., Brown P. D., East N., Crimmin M. J., Balkwill F. R. (1993). A synthetic matrix metalloproteinase inhibitor decreases tumor burden and prolongs survival of mice bearing human ovarian carcinoma xenografts.. Cancer Res.

[OCR_00550] Docherty A. J., Lyons A., Smith B. J., Wright E. M., Stephens P. E., Harris T. J., Murphy G., Reynolds J. J. (1985). Sequence of human tissue inhibitor of metalloproteinases and its identity to erythroid-potentiating activity.. Nature.

[OCR_00557] Firestein G. S., Paine M. M. (1992). Stromelysin and tissue inhibitor of metalloproteinases gene expression in rheumatoid arthritis synovium.. Am J Pathol.

[OCR_00562] Fong K. M., Kida Y., Zimmerman P. V., Smith P. J. (1996). TIMP1 and adverse prognosis in non-small cell lung cancer.. Clin Cancer Res.

[OCR_00566] Hewitt R. E., Leach I. H., Powe D. G., Clark I. M., Cawston T. E., Turner D. R. (1991). Distribution of collagenase and tissue inhibitor of metalloproteinases (TIMP) in colorectal tumours.. Int J Cancer.

[OCR_00571] Hewitt R. E., Powe D. G., Carter G. I., Turner D. R. (1993). Desmoplasia and its relevance to colorectal tumour invasion.. Int J Cancer.

[OCR_00538] Horio Y., Takahashi T., Kuroishi T., Hibi K., Suyama M., Niimi T., Shimokata K., Yamakawa K., Nakamura Y., Ueda R. (1993). Prognostic significance of p53 mutations and 3p deletions in primary resected non-small cell lung cancer.. Cancer Res.

[OCR_00575] Khokha R., Waterhouse P., Yagel S., Lala P. K., Overall C. M., Norton G., Denhardt D. T. (1989). Antisense RNA-induced reduction in murine TIMP levels confers oncogenicity on Swiss 3T3 cells.. Science.

[OCR_00580] Liotta L. A., Stetler-Stevenson W. G. (1991). Tumor invasion and metastasis: an imbalance of positive and negative regulation.. Cancer Res.

[OCR_00584] Muraoka K., Nabeshima K., Murayama T., Biswas C., Koono M. (1993). Enhanced expression of a tumor-cell-derived collagenase-stimulatory factor in urothelial carcinoma: its usefulness as a tumor marker for bladder cancers.. Int J Cancer.

[OCR_00590] Newell K. J., Witty J. P., Rodgers W. H., Matrisian L. M. (1994). Expression and localization of matrix-degrading metalloproteinases during colorectal tumorigenesis.. Mol Carcinog.

[OCR_00595] Nomura S., Hogan B. L., Wills A. J., Heath J. K., Edwards D. R. (1989). Developmental expression of tissue inhibitor of metalloproteinase (TIMP) RNA.. Development.

[OCR_00600] Pavloff N., Staskus P. W., Kishnani N. S., Hawkes S. P. (1992). A new inhibitor of metalloproteinases from chicken: ChIMP-3. A third member of the TIMP family.. J Biol Chem.

[OCR_00605] Poulsom R., Pignatelli M., Stetler-Stevenson W. G., Liotta L. A., Wright P. A., Jeffery R. E., Longcroft J. M., Rogers L., Stamp G. W. (1992). Stromal expression of 72 kda type IV collagenase (MMP-2) and TIMP-2 mRNAs in colorectal neoplasia.. Am J Pathol.

[OCR_00611] Pyke C., Ralfkiaer E., Tryggvason K., Danø K. (1993). Messenger RNA for two type IV collagenases is located in stromal cells in human colon cancer.. Am J Pathol.

[OCR_00616] Senior P. V., Critchley D. R., Beck F., Walker R. A., Varley J. M. (1988). The localization of laminin mRNA and protein in the postimplantation embryo and placenta of the mouse: an in situ hybridization and immunocytochemical study.. Development.

[OCR_00626] Stetler-Stevenson W. G., Krutzsch H. C., Liotta L. A. (1989). Tissue inhibitor of metalloproteinase (TIMP-2). A new member of the metalloproteinase inhibitor family.. J Biol Chem.

[OCR_00622] Stetler-Stevenson W. G. (1990). Type IV collagenases in tumor invasion and metastasis.. Cancer Metastasis Rev.

[OCR_00630] Uría J. A., Ferrando A. A., Velasco G., Freije J. M., López-Otín C. (1994). Structure and expression in breast tumors of human TIMP-3, a new member of the metalloproteinase inhibitor family.. Cancer Res.

[OCR_00635] Witkiewicz H., Bolander M. E., Edwards D. R. (1993). Improved design of riboprobes from pBluescript and related vectors for in situ hybridization.. Biotechniques.

[OCR_00640] Zeng Z. S., Cohen A. M., Zhang Z. F., Stetler-Stevenson W., Guillem J. G. (1995). Elevated tissue inhibitor of metalloproteinase 1 RNA in colorectal cancer stroma correlates with lymph node and distant metastases.. Clin Cancer Res.

[OCR_00647] Zeng Z. S., Guillem J. G. (1995). Distinct pattern of matrix metalloproteinase 9 and tissue inhibitor of metalloproteinase 1 mRNA expression in human colorectal cancer and liver metastases.. Br J Cancer.

